# Supplementary material for: The effects of quality of evidence communication on perception of public health information about COVID-19: Two randomised controlled trials
Source: PLoS One. 2021 Nov 17;16(11):e0259048. doi: 10.1371/journal.pone.0259048 (PMC8598038; doi:10.1371/journal.pone.0259048)
Supplement: Attachment — Submitted filename: Response to Reviewers.docx [file pone.0259048.s003.docx]

**Response to Reviewers**

Reviewers' comments:

Reviewer's Responses to Questions

**Comments to the Author**

1. If the authors have adequately addressed your comments raised in a previous round of review and you feel that this manuscript is now acceptable for publication, you may indicate that here to bypass the “Comments to the Author” section, enter your conflict of interest statement in the “Confidential to Editor” section, and submit your "Accept" recommendation.

Reviewer #3: All comments have been addressed

2. Is the manuscript technically sound, and do the data support the conclusions?

The manuscript must describe a technically sound piece of scientific research with data that supports the conclusions. Experiments must have been conducted rigorously, with appropriate controls, replication, and sample sizes. The conclusions must be drawn appropriately based on the data presented.

Reviewer #3: Yes

3. Has the statistical analysis been performed appropriately and rigorously?

Reviewer #3: Yes

4. Have the authors made all data underlying the findings in their manuscript fully available?

The [PLOS Data policy](http://www.plosone.org/static/policies.action#sharing) requires authors to make all data underlying the findings described in their manuscript fully available without restriction, with rare exception (please refer to the Data Availability Statement in the manuscript PDF file). The data should be provided as part of the manuscript or its supporting information, or deposited to a public repository. For example, in addition to summary statistics, the data points behind means, medians and variance measures should be available. If there are restrictions on publicly sharing data—e.g. participant privacy or use of data from a third party—those must be specified.

Reviewer #3: Yes

5. Is the manuscript presented in an intelligible fashion and written in standard English?

PLOS ONE does not copyedit accepted manuscripts, so the language in submitted articles must be clear, correct, and unambiguous. Any typographical or grammatical errors should be corrected at revision, so please note any specific errors here.

Reviewer #3: Yes

6. Review Comments to the Author

Please use the space provided to explain your answers to the questions above. You may also include additional comments for the author, including concerns about dual publication, research ethics, or publication ethics. (Please upload your review as an attachment if it exceeds 20,000 characters)

Reviewer #3: This paper is well written, the topic is interesting, and the results seem correct; the work is acceptable. I make some recommendations for minor revisions. Besides these issues, the work appears methodologically sound and is well written.

Thank you for taking the time to review our paper and for the positive feedback.

##1
Authors should explain details about limitation of their survey and analysis.

We have added some more details on study limitations to the discussion.

The revised limitations section now reads:

“This study is limited in that it tested only an online population in the US (albeit quota sampled), and only one health intervention. Further research could broaden this population and context, for example, by using true probability samples, collecting data in multiple countries, engaging in field work, and testing a range of public health interventions. A further limitation is that the quality of evidence information provided in this research was a simple indication of the level, without further details as to the exact reasons for the rating. It would be useful to examine the effects of providing greater nuance and detail on the quality rating, in addition to the effects of adding an explanation for recommendations despite low quality of evidence as outlined above. Furthermore, our work only tested the provision of quality of evidence information in a text format. It is conceivable that providing a quality of evidence label in, for example, a graphical format akin to star ratings, might have a different effect. Lastly, our studies were designed to test overall effects across a broad population. Understanding potential differential effects on different subgroups of the population, such as low and high numeracy individuals, would help to complement knowledge on the effects of quality of evidence communication more broadly, and we thus encourage further research to investigate these relationships more deeply.

As mentioned in footnote 3, the images shown to participants showed an icon array that had had some of its icons cropped erroneously. This error was consistent across all studies and conditions and hence unlikely to introduce systematic bias that would affect our results in study 1. In study 2, where we tested in addition a difference in presentation format (with and without icon array display), a bias could have been introduced if participants noticed the varying amounts of light grey icons in the two icon array displays and were confused by it. We hence coded the free text responses that participants provided in both studies to identify any comments about the icon arrays. For neither study were there comments relating to confusion about the icon arrays. It therefore seems likely that participants did not notice the error, and we do not expect any influence of it on our observed effects.”

##2
Figure captions need to be improved (Author can explain in detail).

Thank you for suggesting to improve the figure captions. We agree that providing the reader with more details will aid the reader in understanding the figures fast without needing to refer to the methods section. We have elaborated on the text and explanations given in all figure captions.

##3
Introduction should be improved by adding more recent and related works. For example,

Hypothetical assessment of efficiency, willingness-to-accept and willingness-to-pay for dengue vaccine and treatment: a contingent valuation survey in Bangladesh, Human vaccine and Immunotherapeutics, DOI: 10.1080/21645515.2020.1796424 (2020).

Evolutionary game theory modelling to represent the behavioural dynamics of economic shutdowns and shield immunity in the COVID-19 pandemic. R. Soc. Open Sci. 7: 201095. <http://dx.doi.org/10.1098/rsos.201095> (2020).

“Do humans play according to the game theory when facing the social dilemma situation?” A survey study, EVERGREEN, 07(01), 7-14 (2020).

Prosocial behavior of wearing a mask during an epidemic: an evolutionary explanation. Sci Rep 11, 12621 (2021). <https://doi.org/10.1038/s41598-021-92094-2>.

An evolutionary game modeling to assess the effect of border enforcement measures and socio-economic cost: export-importation epidemic dynamics, Chaos, Solitons & Fractals 146, 110918 (2021).

Thank you for the suggestions. Our introduction was initially specifically about studies of quality of evidence communication, and there were no more recent published studies. However, we have taken the reviewer’s suggestion to reframe the introduction to give more background to the specific pandemic situation in which the work was carried out, and agree that this gives the reader more and useful background. This has given us the opportunity to cite some recent and relevant papers on policies and mitigation strategies employed during the pandemic.

7. PLOS authors have the option to publish the peer review history of their article ([what does this mean?](https://journals.plos.org/plosone/s/editorial-and-peer-review-process#loc-peer-review-history)). If published, this will include your full peer review and any attached files.


If you choose “no”, your identity will remain anonymous but your review may still be made public.


**Do you want your identity to be public for this peer review?** For information about this choice, including consent withdrawal, please see our [Privacy Policy](https://www.plos.org/privacy-policy).

Reviewer #3: No

[NOTE: If reviewer comments were submitted as an attachment file, they will be attached to this email and accessible via the submission site. Please log into your account, locate the manuscript record, and check for the action link "View Attachments". If this link does not appear, there are no attachment files.]

While revising your submission, please upload your figure files to the Preflight Analysis and Conversion Engine (PACE) digital diagnostic tool, <https://pacev2.apexcovantage.com/>. PACE helps ensure that figures meet PLOS requirements. To use PACE, you must first register as a user. Registration is free. Then, login and navigate to the UPLOAD tab, where you will find detailed instructions on how to use the tool. If you encounter any issues or have any questions when using PACE, please email PLOS at [figures@plos.org](mailto:figures@plos.org). Please note that Supporting Information files do not need this step.
